# Efficient Gene Knockout in Goats Using CRISPR/Cas9 System

**DOI:** 10.1371/journal.pone.0106718

**Published:** 2014-09-04

**Authors:** Wei Ni, Jun Qiao, Shengwei Hu, Xinxia Zhao, Misha Regouski, Min Yang, Irina A. Polejaeva, Chuangfu Chen

**Affiliations:** 1 College of Life Sciences, Shihezi University, Shihezi, Xinjiang, China; 2 Department of Animal, Dairy and Veterinary Sciences, Utah State University, Logan, Utah, United States of America; 3 College of Animal Science and Technology, Shihezi University, Shihezi, Xinjiang, China; NIH, United States of America

## Abstract

The CRISPR/Cas9 system has been adapted as an efficient genome editing tool in laboratory animals such as mice, rats, zebrafish and pigs. Here, we report that CRISPR/Cas9 mediated approach can efficiently induce monoallelic and biallelic gene knockout in goat primary fibroblasts. Four genes were disrupted simultaneously in goat fibroblasts by CRISPR/Cas9-mediated genome editing. The single-gene knockout fibroblasts were successfully used for somatic cell nuclear transfer (SCNT) and resulted in live-born goats harboring biallelic mutations. The CRISPR/Cas9 system represents a highly effective and facile platform for targeted editing of large animal genomes, which can be broadly applied to both biomedical and agricultural applications.

## Introduction

Targeted genome editing technologies are crucial for basic biology research, development of animal models and improvement of animal traits for agriculture. Zinc finger nucleases [1], [2], transcription activator-like effector nucleases [3], [4] and homing meganucleases [5] have provided powerful tools to induce targeted mutations in the form of small insertions or deletions derived from DNA break repair of nonhomologous end joining (NHEJ) or homologous recombination. These systems, however, require efficient design and time-consuming assembly of nuclease constructs for DNA targeting. Recently, the CRISPR (clustered regularly interspaced short palindromic repeats)/Cas9 system has been demonstrated as an alternative strategy for precise gene editing [6], [7].

The CRISPR system, as an adaptive immune system in bacteria and archaea, uses small RNAs and CRISPR-associated (Cas) proteins to defend against invading viruses and plasmids [8], [9]. One of the CRISPR systems in Streptococcus pyogenes has been characterized, which includes an endonuclease Cas9, a CRISPR RNA (crRNA) and a transacting RNA (tracrRNA). Cas9 can be programmed to introduce site-specific DNA double-stranded breaks by providing a single guide RNA (gRNA) chimera consisting of a fusion between crRNA and tracrRNA [6]. The two components of Cas9/gRNA have shown high DNA cleavage activity in cultured cells [6], [7], C. elegans [10], zebrafish [11] mice [12] and pigs [13]. These findings encouraged us to explore the possibility of establishing a Cas9/gRNA-based gene modification platform for large animals.

Genetically modified goats are an important tool for producing valuable therapeutic protein [14]–[16] and studying human diseases as ideal biomedical models [17]–[19]. Recombinant human antithrombin, the first ever therapeutic protein from genetically altered goats, have been approved by the US Food and Drug Administration (FDA) [20]. However, it is costly and time-consuming to produce genetically modified livestock animals using standard homologous recombination gene targeting. Multiple gene modifications are especially challenging as the time and cost increase significantly due to the multiple consecutive animal cloning steps, which are required to target different genes. This limits applications of large animals for biomedicine and basic biology research.

In the present study, we show that Cas9/gRNAs can induce precise mutations with efficiency of 9%–70% in goat primary fibroblasts. A single co-transfection of pooled Cas9/gRNAs enabled isolation of cell colonies carrying simultaneous disruption of four genes with high efficiency. The Cas9/gRNA-modified fibroblasts were subjected to nuclear reprogramming by somatic cell nuclear transfer, resulting in live-born goats carrying single-gene mutation.

## Material and Methods

### Ethics statement

All experiments involving animals were conducted under the protocol approved by the Animal Care and Use Committee of Shihezi University and Utah State University.

### gRNA design and plasmid construction

**Figure 1:**
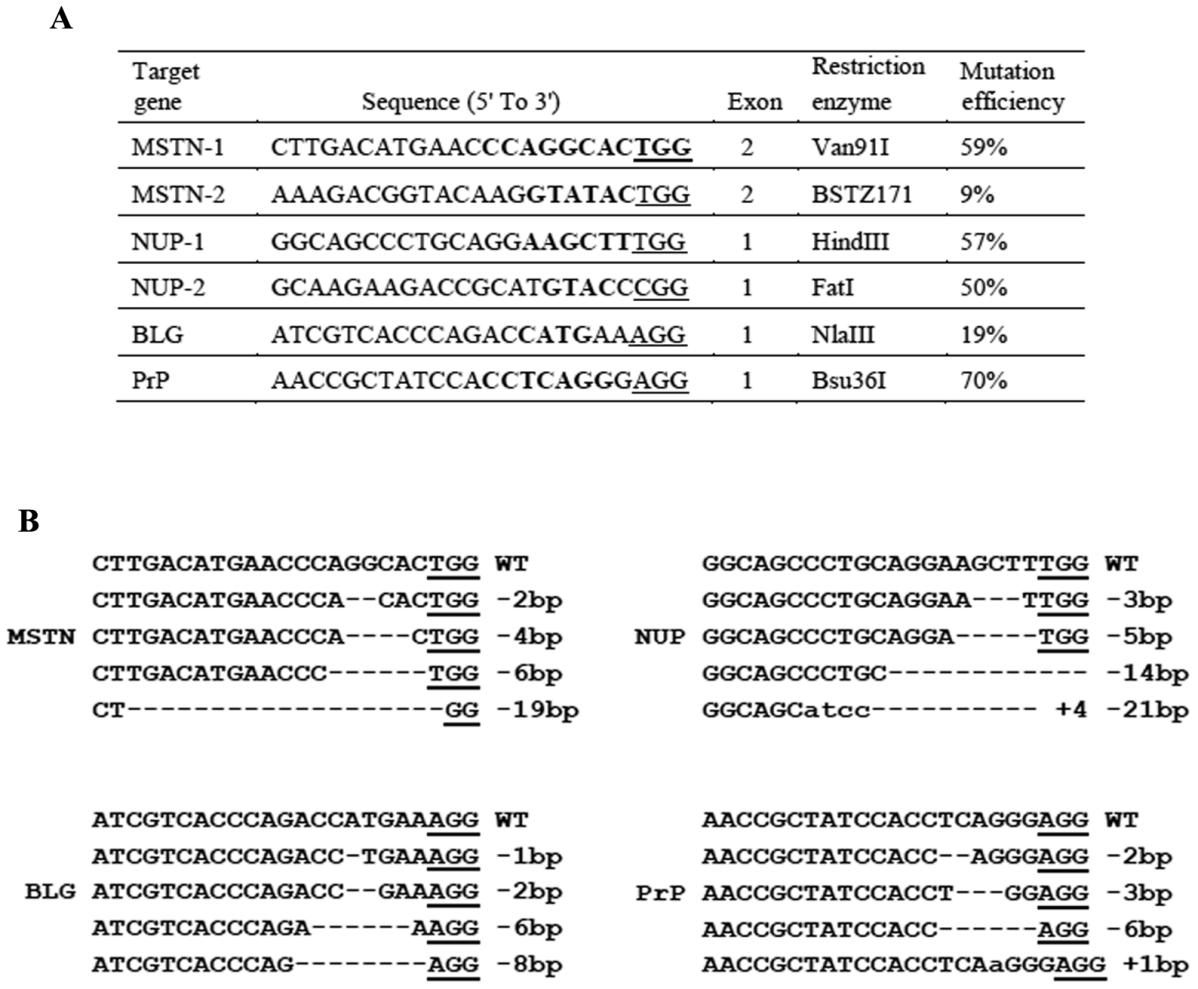
Mutations induced by Cas9/gRNAs in goat fibroblasts. (A) Design and activity of Cas9/gRNAs in goat fibroblasts. The restriction sites in the target regions are bold. The PAM sequence is underlined. (B) Cas9/gRNA-induced mutations in MSTN, PrP, BLG and NUP genes. The sizes of the deletions (−) and insertions (+) are shown to the right of each allele. Insertions are lower case.

Bicistronic expression vector (pX330) expressing both Cas9 and gRNA was generously provided by Dr. Feng Zhang of Broad Institute of MIT and Harvard [6]. gRNAs targeting goat MSTN, NUP, PrP and BLG genes (Figure 1A) were designed as previously described [6]. An extra guanine was added at the 5′ end of gRNA, in which the first nucleotide was not guanine, for more efficient transcription by RNA polymerase III [21]. To facilitate mutation analysis, a restriction enzyme recognition site was incorporated in each target locus (Figure 1A). Site-specific mutations will make the target locus resistant to the restriction enzyme treatment (uncut), which can be detected by restriction fragment length polymorphism (RFLP) assay. The pX330 plasmids were digested with BbsI and gel purified using the Gel Extraction Kit (Qiagen). A pair of oligos for each targeting site (Table S1) were annealed and ligated into linearized pX330 vector for generating gRNA-expressing plasmid.

### Cell culture and transfection

Goat fetal fibroblasts were isolated as described previously [22] and cultured in DMEM supplemented with 15% FBS, 1% sodium pyruvate and 1% penicillin streptomycin to achieve 80–90% confluency on the day of transfection. Cells were transfected with a plasmid (2 µg) expressing both Cas9 and gRNA targeting MSTN-1 (single targeting), two plasmids (2 µg of each plasmids) expressing Cas9 and gRNA targeting MSTN-1 and PrP genes (double targeting), or four plasmids (2 µg of each plasmids) expressing Cas9 and gRNA targeting MSTN-1, PrP, BLG and NUP-1 genes (quadruple targeting) using Nucleofector (Amaxa) according to the manufacturer's protocol. 72 h after transfection, cells were collected for RFLP assay (Mutation frequency analysis) or were seeded individually into 96-well plates for isolating single cell colonies. Single cell-derived colonies were harvested after 10–14 days of culture.

### RFLP assay and DNA sequencing

Genomic DNA was isolated from treated and wild-type cell colonies, or ear biopsies of cloned and wild-type goats. PCR was performed on 500 ng of genomic DNA using specific primers against MSTN, BLG, PrP and NUP (Table S1). 10 µL of PCR products were digested with Van91I, Bsu36I, NlaIII or HindIII, respectively. Digested DNA was visualized by agarose gel (1.5–2.0%). Mutation frequencies were calculated as previously described [23]. For DNA sequencing, uncleaved bands from digested DNA were gel purified and subjected to TA cloning. Fifteen transformed colonies (E. coli strain) for each group were randomly picked up and sequenced. DNA mutations were identified by sequence alignment between sequenced allele and wild type allele.

### Somatic cell nuclear transfer

Goat somatic cell nuclear transfer was performed as we described previously [24]. Briefly, ovaries were collected from abattoir and transported to our laboratory within 4 h after slaughter. Cumulus-oocyte complexes (COCs) were aspirated from 2 to 5 mm follicles with PBS (containing 5% FCS) by using a 5 ml syringe fitted with a 20-gauge needle. The COCs were cultured in maturation medium at 38.5°C in a humidified atmosphere for 22–24 h. Cumulus cells were removed by exposure to 1 mg/mL hyaluronidase. Oocytes with a first polar body were enucleated manually in the presence of 7.5 µg/ml of cytochalasin B. A single intact donor cell was injected into the perivitelline space and placed adjacent to the recipient viteline membrane. After injection, reconstructed embryos were transferred into an electrical fusion chamber overlaid with Zimmermann's fusion medium. Cell fusion was induced with two direct current pulses (1.0 kV/cm, 60 µs, 1s apart). Fused reconstructed embryos were further activated in 5 µM ionomycin for 5 min, followed by exposure to 1.9 mM 6-dimethylaminopurine (DMAP) in synthetic oviduct fluid with amino acids (SOFaa) for 4 h. Following activation, embryos were then transferred and cultured in SOFaa. Embryos that developed to the 2 to 4-cell stages were surgically transferred into synchronized recipient does (10–15 embryos per recipient). Pregnancies were confirmed by ultrasound scanning using a trans-abdominal linear probe on day 45. Wild-type control goats were produced by normal sexual reproduction.

### Western blotting

Protein extracts were taken from biceps brachii muscles of cloned and wild type goats. To raise antibodies against goat MSTN, the C-terminal region of goat MSTN (amino acids 266–375) was expressed in bacteria, purified with a nickel column and then used as antigen to immunize rabbits. Anti-goat MSTN monoclonal antibody (1∶2000 dilution) and 1∶1000 dilution of a mouse anti-actin antibody (Sigma, A4700) were used for the western blot analysis. Western blotting was performed as previously reported [25]. The band intensities were estimated by densitometry and corrected by the actin band intensities.

### Analysis of potential off targets

The potential off-target sites were selected based on the following rules: (1) the protospacer-adjacent motif (PAM) sequence is NGG; (2) the homology with the 12 base “seed sequence” at the 3′ end of the gRNA [6]. We searched whole goat genome using BLAST tool (http://www.ncbi.nlm.nih.gov/genome/10731) against 13 bp of MSTN-1 gRNA seed sequence. Eight potential off targets were found in goat genome for MSTN-1 gRNA. These potential off-target sites were amplified from all cloned goats and subjected to TA cloning. Fifteen transformed colonies (E. coli strain) for each potential off-targets were randomly picked up and sequenced.

## Results

### Assessment of Cas9/gRNA activity in goat primary cells

To test Cas9/gRNA activity in goat primary fibroblasts, we designed six gRNAs targeting myostatin (MSTN), nucleoporin 155 (NUP), prion protein (PrP), and beta-lactoglobulin (BLG), respectively (Figure 1A). Each bicistronic plasmid expressed Cas9 and one of the gRNAs. The plasmids targeting each gene were respectively transfected into goat fibroblasts, and their genome modification efficiency was determined at day 3 using restriction fragment length polymorphism (RFLP) assay (see Methods). Surprisingly, all Cas9/gRNAs showed high cleavage activity (9%–70%) in goat fibroblasts (Figure 1A and Figure S1). DNA sequencing further confirmed that some small deletions and insertions (indels) were introduced into the gRNA target regions (Figure 1B).

### Simultaneous disruption of four genes in single cell-derived colonies

**Figure 2:**
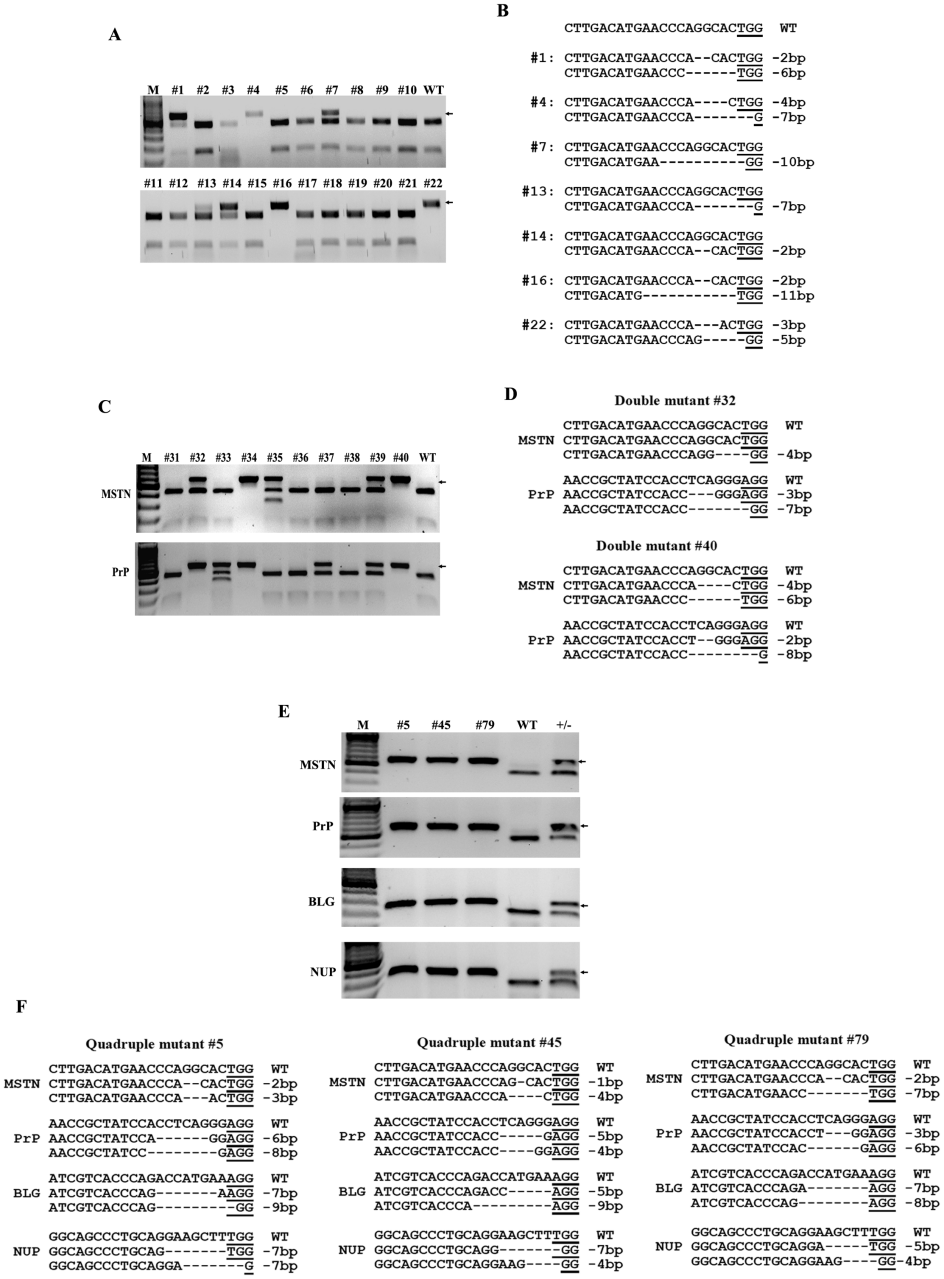
Single-, double- and quadruple-gene targeting in goat primary fibroblasts. (A) Genotyping of MSTN-mutant colonies by the RFLP assay. MSTN mutations in cell colony make the target locus resistant to Van91I treatment (arrowhead). Colonies #1, #7, #13 and #14 carry mono-allelic mutations of MSTN. Colonies #4, #16 and #22 carry biallelic mutations of MSTN. (B) DNA sequence of mutated alleles from MSTN-mutant colonies. Sequence of seven mutant colonies is shown. The PAM sequence is underlined. (C) Genotyping of MSTN/PrP double-mutant colonies by the RFLP assay. Analysis of colonies #31 to #40 is shown. MSTN/PrP double mutations in cell colony make the target locus resistant to Van91I and Bsu36I treatment (arrowhead). Colonies #32, #34, #39 and #40 carry mutations in both genes. Moreover, colonies #34 and #40 have biallelic mutations in both genes. (D) DNA sequence of mutated alleles from MSTN/PrP double-mutant colony #32 and #40. (E) MSTN/PrP/BLG/NUP quadruple-mutant colonies were confirmed by the RFLP assay. Colonies #5, #45 and #79 had biallelic mutations in all four target genes (arrowhead). Wild-type (WT) and monoallele-mutant (+/−) cells were used as controls. (F) The sequence of eight mutant alleles from quadruple-mutant colonies #5, #45 and #79.

Isolation of single cell-derived colonies is required for developing methods of Cas9/gRNA-mediated targeting in livestock by animal cloning. We first tried to isolate indel-mutant colonies from MSTN gRNA transfected fibroblasts. Of 22 colonies by the RFLP assay, seven carried mutations in the MSTN gene and three of them had biallelic mutations of MSTN (Table 1 and Figure 2A). DNA sequencing further showed that these cell colonies included some small deletions in the MSTN gene (Figure 2B).

**Table 1:** Cas9/gRNA-mediated multiple gene disruptions in goat fibroblasts.

Target gene	Colonies tested	No. mutant colonies (%)			
		Single (%)	Double (%)	Triple (%)	Quadruple (%)
MSTN	22	7 (32)	-	-	-
MSTN/PrP	45	19 (42)	9 (20)	-	-
MSTN/BLG/PrP/NUP	107	35 (32)	30 (28)	9 (8)	3 (2)

The high efficiencies of Cas9/gRNAs inspired us to attempt targeting two genes simultaneously in goat primary fibroblasts. MSTN Cas9/gRNA and PrP Cas9/gRNA were co-transfected into goat fibroblasts and 9 out of 45 colonies were identified to carry mutations in both genes by the RFLP assay (Table 1 and Figure 2C). Among colonies with double-gene mutations, 55% (5 of 9) of the mutants had mutations in all four alleles of these two genes (Figure 2D and Figure S2).

We next tested the efficiency of Cas9/gRNAs disrupting four genes simultaneously by co-transfecting pooled Cas9/gRNAs targeting MSTN, BLG, PrP and NUP genes. The RFLP assay showed that 3 out of 107 colonies had biallelic mutations in all four genes (Table 1 and Figure 2E). DNA sequencing of three quadruple-mutant colonies further confirmed that small deletions were present in all eight alleles of four genes (Figure 2F).

### Nuclear transfer to produce Cas9/gRNA-modified goats

**Figure 3:**
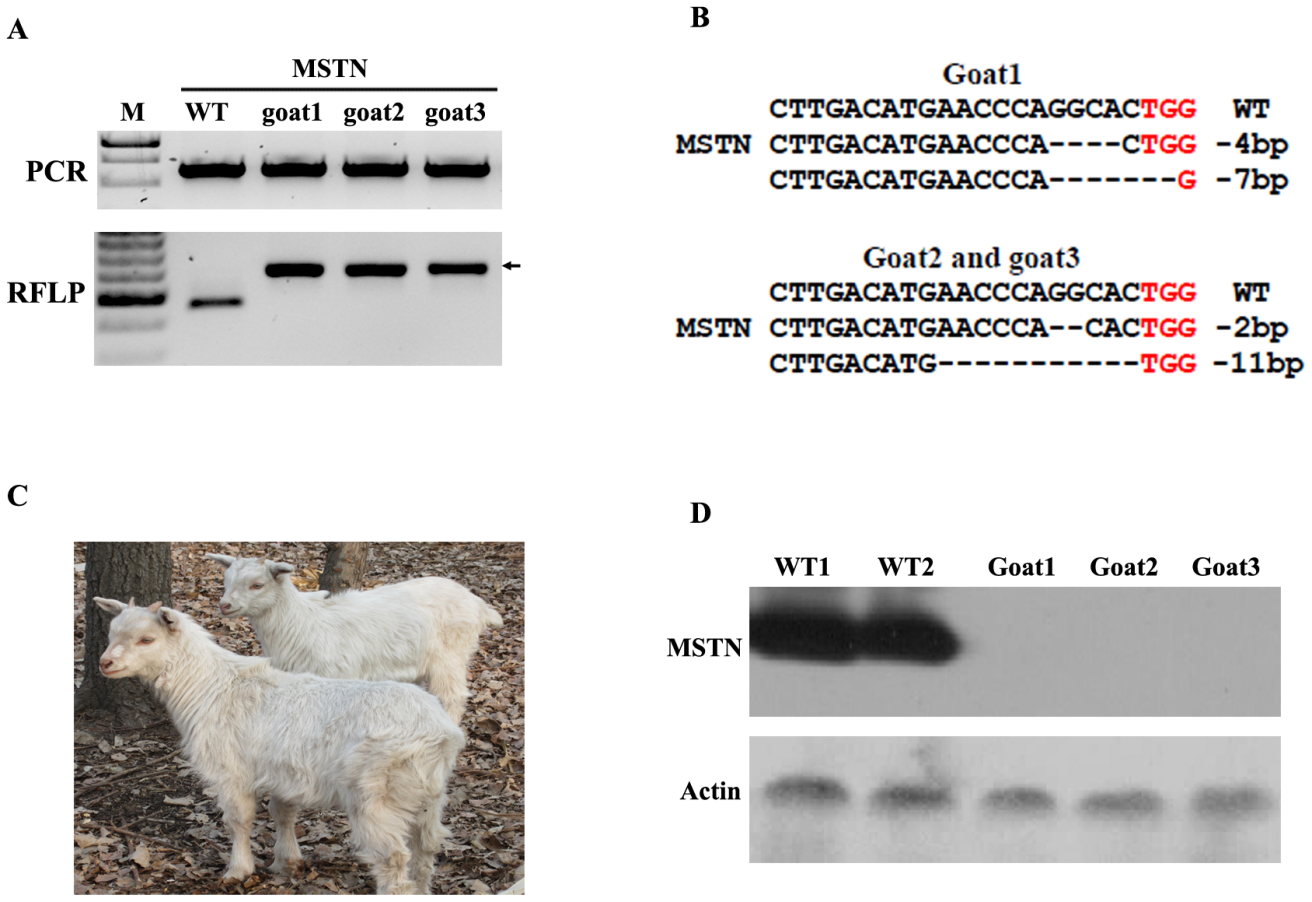
Cloned goats carrying MSTN biallelic mutations. (A) RFLP assay of cloned goats. Goat1 to goat3 derived from MSTN-modified cells. The PCR products spanning the MSTN target site from each goat are shown in the upper panel. The PCR products were used for RFLP assay (lower panel). MSTN mutations in cloned goat make the target locus resistant to Van91I treatment (arrowhead). (B) Sequence analysis confirming genotypes of three cloned goats. The PAM sequence is shown in red. Wild-type (WT) sequence is shown above. (C) Cloned goats carrying MSTN biallelic mutation. (D) Western blotting for detecting MSTN expression. WT1 and WT2: wild-type goats; goat1, goat2 and goat3: three MSTN mutant goats.

Cell colonies with MSTN biallelic mutations (MSTN-KO) were used for nuclear transfer. MSTN-KO colonies (MSTN-KO4 and MSTN-KO16) yielded seven pregnancies from 21 transfers. Three pregnancies were maintained to term, resulting in three live-born goats (Goat1 to goat3) (Table 2). The RFLP assay and DNA sequencing showed that all three goats carried biallelic mutations in the MSTN gene (Figure 3A and Figure 3B). Goat3 died 3 days after birth and the remaining two are currently healthy at over 3 months of age. The early deaths of cloned goats were related to physical defect as a result of SCNT. MSTN expression in the muscles of cloned goats was detected by using western blotting. MSTN expression was not observed in cloned goats (Figure 3D), which confirmed disruption of MSTN expression resulting from frameshift mutations.

**Table 2:** Summary of SCNT results.

Cell colonies	No. embryos transferred	No. recipient goats	No. pregnancy (D45) (%)	No. goats born (alive)
MSTN-KO4	134	10	3 (30)	1 (1)
MSTN-KO16	135	11	4 (36)	2 (1)

### Off-target analysis of Cas9-mediated mutation

To assess potential off-targets of Cas9/gRNA in goats, we searched for other genomic sequences that could potentially be targeted by MSTN Cas9/gRNA. Eight potential off targets were found in goat genome for MSTN gRNA1. These potential off-target sites were analyzed by DNA sequencing, but no unwanted mutation occurred at these genomic sites in all cloned goats (Table S2). These results indicate that the 12 base “seed sequence” at the 3′ end of the gRNA confers target specificity in goat cells, similar to a study in human cells [26]. However, we cannot exclude other potential off-target events following as yet unidentified rules, and comprehensive off-target profiling need be performed in the future.

## Discussion

In this study, we demonstrate for the first time that Cas9/gRNA-mediated gene knockout approach is highly efficient in goats resulting in successful generation of cloned goats with biallelic mutations. Recently, Hai et al. reported generation of single-gene knockout pigs by zygote injection of CRISPR/Cas system [13]. Although direct modification of zygotic genomes may have some advantages, the strategy can result in mosaic or hypomorphic mutation of injected animals [27]–[31], for which mutation may fail to transmit to offspring [27] and breeding will need to be further performed for obtaining homozygous (nonmosaic) animals [29]. In contrast, somatic cell modification followed by SCNT allow the isolation of mutant cells before the expense of animal production and ensure producing animals with expected gene modifications. We designed six Cas9/gRNAs targeting four different genes, and all these Cas9/gRNAs showed high mutation efficiency in goat primary fibroblasts (Figure 1). We did not observe any abnormal growth or morphological changes in Cas9/gRNA-treated cells. Cloned embryo from Cas9/gRNA-modified cells resulted in normal pregnancy and birth of cloned goats (Table 2). The cloning efficiency was 1.1%, similar to the result obtained with transgenic fibroblasts previously published by other groups [32], [33]. Our results suggest that the CRISPR/Cas9 system combined with SCNT technology is a highly efficient strategy for targeted editing of large animal genomes.

We also demonstrate the feasibility of Cas9/gRNA-mediated multiple gene modifications in primary cells. A single co-transfection using two and four Cas9/gRNAs enabled isolation of cell colonies harboring double and quadruple disruptions in 20% and 2% of colonies, respectively, which may not be easily achieved with ZFNs and TALENs technologies. The one-step generation of multiplex mutations in large animals marks a significant improvement over traditional sequential targeting, a process necessitating multiple rounds of animal cloning to target different genes. For example, Kuroiwa et al. reported that it took upwards of 21.5 months or longer for producing double-gene knockout cattle [34]. Furthermore, due to high efficiency of CRISPR/Cas9 system, cell colonies carrying multiple gene mutations can be isolated by limiting dilution and is free of selection maker, which avoids further removal of selection marker by recloning or intercrossing [35].

In conclusion, we show for the first time that the CRISPR/Cas9 mediated genome editing can be efficiently accomplished in goats. Cas9/gRNA can be easily engineered against almost all endogenous genes within a 3-day period of time, and goats carrying multiplex mutations can be generated within 5 months (gestation length 150–155 days). Cas9/gRNA-mediated gene targeting demonstrated in this study can be used for other livestock species, which will contribute to advancing transgenic applications of large animal in biomedical and agricultural sciences.

## Supporting Information

Figure S1 **RFLP assay for detecting Cas9/gRNA-mediated mutations.** PCR products from MSTN, NUP, BLG and PrP Cas9/gRNA-treated cells were digested with Van91I, BSTZ171, HindIII, FatI, NlaIII, Bsu36I, respectively; WT: PCR products from wild-type cells were digested with Van91I, BSTZ171, HindIII, FatI, NlaIII, Bsu36I, respectively.
(PDF)

Figure S2 **Genotyping of double-mutant colonies by DNA sequencing.** Of nine colonies with MSTN/PrP double mutations, five colonies had biallelic mutations of both genes. The PAM sequence is labeled in red.
(PDF)

Table S1 **Oligonucleotides used in this study.**
(PDF)

Table S2 **Potential off targets of MSTN gRNA1.**
(PDF)
