# Supplementary material for: Efficient Gene Knockout in Goats Using CRISPR/Cas9 System
Source: PLoS One. 2014 Sep 4;9(9):e106718. doi: 10.1371/journal.pone.0106718 (PMC4154755; doi:10.1371/journal.pone.0106718)
Supplement: Figure S1 — RFLP assay for detecting Cas9/gRNA-mediated mutations. PCR products from MSTN, NUP, BLG and PrP Cas9/gRNA-treated cells were digested with Van91I, BSTZ171, HindIII, FatI, NlaIII, Bsu36I, respectively; WT: PCR products from wild-type cells were digested with Van91I, BSTZ171, HindIII, FatI, NlaIII, Bsu36I, respectively. (PDF) [file pone.0106718.s001.pdf]

Figure S1

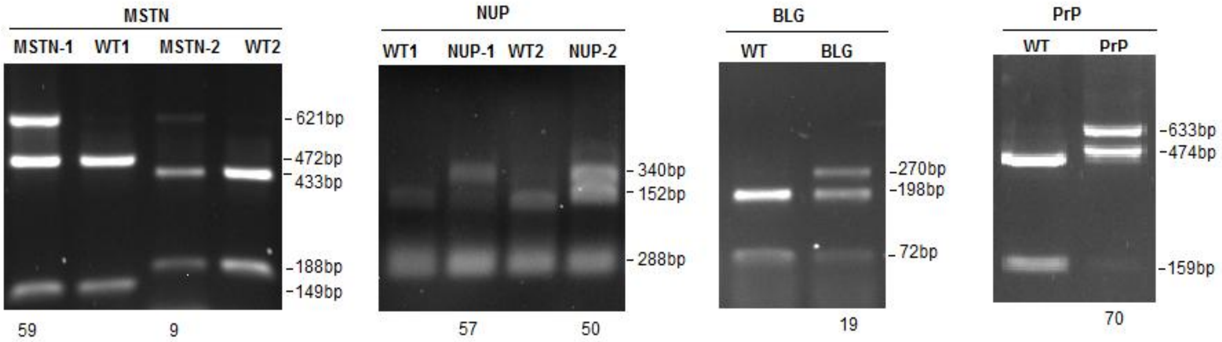

Figure S1. RFLP assay for detecting Cas9/gRNA-mediated mutations. PCR products from MSTN, NUP, BLG and PrP Cas9/gRNA-treated cells were digested with Van91I, BSTZ171, HindIII, FatI, NlaIII, Bsu36I, respectively; WT: PCR products from wild-type cells were digested with Van91I, BSTZ171, HindIII, FatI, NlaIII, Bsu36I, respectively.
