# Supplementary material for: Efficient Gene Knockout in Goats Using CRISPR/Cas9 System
Source: PLoS One. 2014 Sep 4;9(9):e106718. doi: 10.1371/journal.pone.0106718 (PMC4154755; doi:10.1371/journal.pone.0106718)
Supplement: Figure S2 — Genotyping of double-mutant colonies by DNA sequencing. Of nine colonies with MSTN/PrP double mutations, five colonies had biallelic mutations of both genes. The PAM sequence is labeled in red. (PDF) [file pone.0106718.s002.pdf]

Figure S2

MSTN/PrP double-mutant colonies (1 to 5 have biallelic mutations of both genes)

```
1
       CTTGACATGAACCCAGGCACTGG   WT
MSTN   CTTGACATGAACCCAG----TGG   -4
       CTTGACATGAACCC------TGG   -6

       AACCGCTATCCACCTCAGGGAGG   WT
PrP    AACCGCTATCCACCT------GG   -6
       AACCGCTATCCACC------AGG   -6

2
       CTTGACATGAACCCAGGCACTGG   WT
MSTN   CTTGACATGAACCCAG---CTGG   -3
       CTTGACATGAACCCAGGC---GG   -3

       AACCGCTATCCACCTCAGGGAGG   WT
PrP    AACCGCTATCCACCTCA---AGG   -3
       AACCGCTATCCACCTC-----GG   -5

3
       CTTGACATGAACCCAGGCACTGG   WT
MSTN   CTTGACATGAACCCA----CTGG   -4
       CTTGACATGAACCC------TGG   -6

       AACCGCTATCCACCTCAGGGAGG   WT
PrP    AACCGCTATCCACCT--GGGAGG   -2
       AACCGCTATCCACC--------G   -8

4
       CTTGACATGAACCCAGGCACTGG   WT
MSTN   CTTGACATGAACCCA----CTGG   -4
       CTTGACATGAACCCAGG----GG   -4

       AACCGCTATCCACCTCAGGGAGG   WT
PrP    AACCGCTATCCACC------AGG   -6
       AACCGCTATCCACCT--------   -9

5
       CTTGACATGAACCCAGGCACTGG   WT
MSTN   CTTGACATGAACCCAGGC--TGG   -2
       CTTGACATGAACCCAGGC--TGG   -2

       AACCGCTATCCACCTCAGGGAGG   WT
PrP    AACCGCTATCCAC--------GG   -8
       AACCGCTATCCACCT--GGGAGG   -2

6
       CTTGACATGAACCCAGGCACTGG   WT
MSTN   CTTGACATGAACCCAGGCACTGG   WT
       CTTGACATGAACCCAGG----GG   -4

       AACCGCTATCCACCTCAGGGAGG   WT
PrP    AACCGCTATCCACC---GGGAGG   -3
       AACCGCTATCCACC-------GG   -7

7
       CTTGACATGAACCCAGGCACTGG   WT
MSTN   CTTGACATGAACCC---------   -9
       CTTGACATGAACCCAGGC-----   -5

       AACCGCTATCCACCTCAGGGAGG   WT
PrP    AACCGCTATCCACCT------GG   -6
       AACCGCTATCCACCTCAGGGAGG   WT

8
       CTTGACATGAACCCAGGCACTGG   WT
MSTN   CTTGACATGAACCCAGGC-----   -5
       CTTGACATGAACCCAGGCACTGG   WT

       AACCGCTATCCACCTCAGGGAGG   WT
PrP    AACCGCTATCCACC------AGG   -6
       AACCGCTATCCACCTCA--GAGG   -2

9
       CTTGACATGAACCCAGGCACTGG   WT
MSTN   CTTGACATGAACCCAGGCACTGG   WT
       CTTGACATGAACCCAGGC-----   -6

       AACCGCTATCCACCTCAGGGAGG   WT
PrP    AACCGCTATCCAC-------AGG   -7
       AACCGCTATCCACCTCAGGGAGG   WT
```

Figure S2. Genotyping of double-mutant colonies by DNA sequencing. Of nine colonies with MSTN/PrP double mutations, five colonies had biallelic mutations of both genes. The PAM sequence is labeled in red.
