# Supplementary material for: Efficient Gene Knockout in Goats Using CRISPR/Cas9 System
Source: PLoS One. 2014 Sep 4;9(9):e106718. doi: 10.1371/journal.pone.0106718 (PMC4154755; doi:10.1371/journal.pone.0106718)
Supplement: Table S1 — Oligonucleotides used in this study. (PDF) [file pone.0106718.s003.pdf]

Table S1. Oligonucleotides used in this study

Oligonucleotides used for constructing gRNA expression vector

Target gene	Sequence (5' to 3')
MSTN-1	F: CACCGCTTGACATGAACCCAGGCAC<br>R: AAACGTGCCTGGGTTCATGTCAAGC
MSTN-2	F: CACCGAAAGACGGTACAAGGTATAC<br>R: AAACGTATACCTTGTACCGTCTTTC
NUP-1	F: CACCGGCAGCCCTGCAGGAAGCTT<br>R: AAACAAGCTTCCTGCAGGGCTGCC
NUP-2	F: CACCGCAAGAAGACCGCATGTACC<br>R: AAACGGTACATGCGGTCTTCTTGC
PrP	F: CACCGAACCGCTATCCACCTCAGGG<br>R: AAACCCCTGAGGTGGATAGCGGTTC
BLG	F: CACCGATCGTCACCCAGACCATGAA<br>R: AAACTTCATGGTCTGGGTGACGATC

PCR primers used for the RFLP assay

Gene	Primer sequence (5’- 3’)	Tm	Size of PCR amplicon (bp)
MSTN	F: CTGGAAAGGAAGTAGGCTGCTC<br>R: GTCCTTCTTCTCCTGGTTCTGG	64	621
BLG	F: TGAAGTGCCTCCTGCTTGCCCT<br>F: AGGAGAAGCCAGCCTGGCCGA	70	307
PrP	F: TGAAAAGCCACATAGGCAGTT<br>R: CCACTCCCTCCATTATCTTGA	58	633
NUP	F: TGACTTTCTCCGGTTGCTGTT<br>R: CTGGAGAAGGGAATGGCAAC	64	340
