# Supplementary material for: Efficient Gene Knockout in Goats Using CRISPR/Cas9 System
Source: PLoS One. 2014 Sep 4;9(9):e106718. doi: 10.1371/journal.pone.0106718 (PMC4154755; doi:10.1371/journal.pone.0106718)
Supplement: Table S2 — Potential off targets of MSTN gRNA1. (PDF) [file pone.0106718.s004.pdf]

Table S2. Potential off targets of MSTN gRNA1

Site	Location	Seed sequence-PAM	Strand	Gene	Off target
MSTN1	Chr1:140973080-140973095	tgattccTGAACCCAGGCACTGG	+	19kb 5' of alpha-crystallin A chain	NO
MSTN2	Chr4: 91704465-91704480	cagggacTGAACCCAGGCACTGG	-	8kb 5' of centrosomal protein	NO
MSTN3	Chr9: 26023253-26023268	caggcatTGAACCCAGGCACTGG	-	35kb 5' of adapter protein CIKS isoform X2	NO
MSTN4	Chr13: 38111258-38111273	ccaggatTGAACCCAGGCACTGG	-	N-alpha-acetyltransferase 20	NO
MSTN5	Chr15:77474108-77474123	taaatttTGAACCCAGGCACTGG	+	0.6kb 3' of ciliary neurotrophic factor	NO
MSTN6	Chr18: 25082460-25082475	cagggatTGAACCCAGGCACTGG	-	probable G-protein coupled receptor 97	NO
MSTN7	Chr21:14407238-14407253	actgaacTGAACCCAGGCACTGG	+	A-kinase anchor protein 13-like	NO
MSTN8	Chr22: 49125303-49125318	cagggacTGAACCCAGGCACTGG	-	protein VPRBP isoform X1	NO
